# Cross-correlation spin noise spectroscopy of heterogeneous interacting spin systems

**DOI:** 10.1038/srep09573

**Published:** 2015-04-30

**Authors:** Dibyendu Roy, Luyi Yang, Scott A. Crooker, Nikolai A. Sinitsyn

**Affiliations:** 1 Theoretical Division, Los Alamos National Laboratory, Los Alamos, NM 87545, USA; 2 Center for Nonlinear Studies, Los Alamos National Laboratory, Los Alamos, NM 87545, USA; 3 National High Magnetic Field Laboratory, Los Alamos National Laboratory, Los Alamos, NM 87545, USA

## Abstract

Interacting multi-component spin systems are ubiquitous in nature and in the laboratory. As such, investigations of inter-species spin interactions are of vital importance. Traditionally, they are studied by experimental methods that are necessarily perturbative: *e.g.*, by intentionally polarizing or depolarizing one spin species while detecting the response of the other(s). Here, we describe and demonstrate an alternative approach based on multi-probe *spin noise spectroscopy*, which can reveal inter-species spin interactions - under conditions of strict thermal equilibrium - by detecting and cross-correlating the stochastic fluctuation signals exhibited by each of the constituent spin species. Specifically, we consider a two-component spin ensemble that interacts via exchange coupling, and we determine *cross-correlations* between their intrinsic spin fluctuations. The model is experimentally confirmed using “two-color” optical spin noise spectroscopy on a mixture of interacting Rb and Cs vapors. Noise correlations directly reveal the presence of inter-species spin exchange, without ever perturbing the system away from thermal equilibrium. These non-invasive and noise-based techniques should be generally applicable to any heterogeneous spin system in which the fluctuations of the constituent components are detectable.

There are numerous natural and engineered systems in which interactions between “spins of different kind” lead to the emergence of new and interesting physics. Notable examples include the interaction between electron spins from different Bloch bands that gives rise to heavy-fermion behavior and Kondo-lattice effects in correlated-electron materials^1^,^2^, the decoherence of solid-state electronic spin qubits by a nuclear spin bath^3^,^4^,^5^,^6^, carrier-mediated ferromagnetism in diluted magnetic semiconductors^7^,^8^, and spin-exchange pumping of noble gas nuclei for enhanced medical imaging^9^. Measurements of inter-species spin interactions are therefore essential to our understanding of many magnetic and spin phenomena.

Where possible, inter-species spin interactions are generally studied by well-developed perturbative experimental methods that, for example, selectively polarize or depolarize one spin species while separately monitoring the influence on the other(s)^10^. However, interaction cross-sections often depend strongly and non-linearly on the non-equilibrium spin polarizations that are experimentally induced^11^. For many spin systems, it may therefore be desirable to measure spin interactions under conditions as close to thermal equilibrium as possible.

As an alternative to conventional perturbation-based techniques for measuring spin and magnetization dynamics, methods for optical *spin noise spectroscopy*^12^,^13^,^14^ have been recently developed in which electron and hole spin dynamics are revealed solely via the passive detection of their intrinsic and random spin fluctuations in thermal equilibrium – *i.e.*, without any polarization, excitation, or pumping. To date, spin noise spectroscopy has been applied to many different *single* species of spins, such as specific alkali atoms^12^,^15^,^16^,^17^,^18^, itinerant electron spins in semiconductors^19^,^20^,^21^,^22^, and localized hole spins in quantum dot ensembles^6^,^23^. These studies have shown that dynamic properties of spin ensembles such as g-factors, relaxation rates and decoherence times are measurable simply by “listening” (typically via optical Faraday rotation) to the system's intrinsic and random spin noise – an approach ensured by the fluctuation-dissipation theorem.

Based on these developments, here we explore whether spin *interactions* between *different* spin ensembles can also be directly revealed and studied – under conditions of strict thermal equilibrium – through their stochastic spin fluctuations alone. We envision a type of experiment shown schematically in Fig. 1, wherein two spin species A and B in thermal equilibrium interact, *e.g.*, by direct spin exchange as depicted^24^,^25^. If the intrinsic spin fluctuations from species A and B can be independently detected, then signatures of spin interactions should appear in the *cross-correlation* of these two spin noise signals. For example, anti-correlations can be expected in the presence of purely spin-exchange coupling, since interactions causing positive spin fluctuations in one species will be accompanied by corresponding negative fluctuations in the other species.

In this paper we show that multi-probe spin noise spectroscopy can indeed reveal inter-species spin-spin interactions by detecting the system's intrinsic stochastic spin fluctuations. We develop a theory for such cross-correlations in heterogeneous spin systems at thermodynamic equilibrium. In particular, we prove a universal sum rule (a “no-go theorem”) that imposes restrictions on such cross-correlators. These results are directly compared to a proof-of-principle experimental study of a well-understood interacting spin system (a mixture of warm Rb and Cs vapors) by applying a new type of “two-color” spin noise spectroscopy^26^, and excellent agreement is found. Thus, we introduce a framework for both theoretical and experimental exploration of a broad class of heterogeneous interacting spin systems by detecting their spin fluctuations in equilibrium.

## Results

### Experimental setup

**Figure 2:**
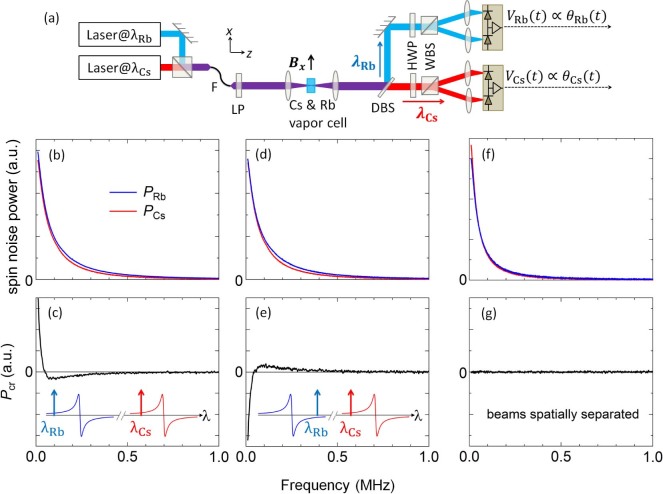
Measuring spin noise power spectra and cross-correlation spectra in a mixture of Rb and Cs atomic vapors. (a) Experimental setup: two probe lasers are detuned by 30–100 GHz from the Rb and Cs D1 transitions (794.98 nm and 894.59 nm, respectively), then combined in a single-mode fiber (F), and focused through the Rb/Cs vapor cell. Random spin fluctuations *S_z_*(*t*) in Rb and Cs impart Faraday rotation (FR) fluctuations *θ*(*t*) on the transmitted probes, which are then separated by a dichroic beam splitter (DBS) and measured by balanced photodiodes. LP: linear polarizer, HWP: half-wave plate, WBS: Wollaston beam splitter. (b) Spin noise power density *P*_Rb_(*ω*) and *P*_Cs_(*ω*) from Rb and Cs spin fluctuations at *B_x_* = 0. (c) The corresponding cross-correlator *P*_cr_(*ω*). (d,e) Similar, but for the case when one probe laser is detuned *above* its D1 transition. Insets: cartoons showing the wavelength-dependent FR amplitude that is induced by polarized ground-state (valence) electrons in Rb and Cs, in the vicinity of their respective D1 transitions; also shown are the relative positions of the probe laser wavelengths *λ*_Rb_ and *λ*_Cs_. (f,g) Corresponding noise and cross-correlation data for the case of spatially-separated probe beams. Note that here *λ*_Rb_ and *λ*_Cs_ are further detuned from their respective D1 transition, giving narrower noise (see text).

To most easily introduce the notion of spin noise spectroscopy and to describe how spin fluctuations are detected and correlated, we first describe the experiment and its results. Figure 2(a) depicts the setup. A 20 mm long glass cell containing both Rb and Cs metal (and 100 Torr of Ar buffer gas) is heated to ~140°C, giving a classical alkali vapor with Rb and Cs particle densities of about 0.6 and 1.4 × 10^14^ cm^−3^ respectively. To independently probe the intrinsic spin fluctuations in both species, we perform spin noise spectroscopy^12^ using *two* linearly-polarized probe lasers with wavelengths *λ*_Rb_ ~ 795.0 nm and *λ*_Cs_ ~ 894.6 nm that are, respectively, tuned close to (but not on) the fundamental D1 ( ^2^*S*_1/2_–^2^*P*_1/2_) optical transitions of Rb and Cs. In fact, *λ*_Rb_ and *λ*_Cs_ are intentionally detuned below their respective D1 transitions (anywhere from 30–100 GHz), to enable detection of the Rb and Cs spin polarization via optical Faraday rotation^27^. This large detuning significantly exceeds the Doppler or pressure broadening or hyperfine splitting of the D1 transitions, ensuring that the probe lasers do not pump or excite the atoms to leading order. Moreover, the large detuning of the probe lasers compared to the pressure broadening of the D1 lines due to the buffer gas (~10 GHz) greatly simplifies the analysis of the data because we can ignore the hyperfine sub-structure of the D1 transition and can effectively consider the Rb and Cs atoms as having simple spin-1/2 magnetic ground states^28^.

The random spin fluctuations of the Rb and Cs valence electrons along the 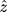 direction – *S*_Rb,*z*_(*t*) and *S*_Cs,*z*_(*t*) – are detected by the optical Faraday rotation (FR) fluctuations *θ*_Rb_(*t*) and *θ*_Cs_(*t*) that they impart on the detuned probe lasers. This detection scheme is made possible by the optical selection rules in alkali atoms, and because FR depends not on absorption constants but rather on the right- and left- circularly polarized indices of refraction of the alkali vapors (*θ* ∝ *n^R^* − *n^L^*), which are dispersive and decay slowly (inversely) with large laser detuning^27^. The two detuned lasers can therefore be regarded as passive, non-perturbing probes of the Rb and Cs vapor's intrinsic spin fluctuations^12^,^17^,^18^,^29^. The FR sensitivity of the lasers to spin fluctuations scales inversely with (and can be adjusted via) the laser detuning from the D1 line.

The two probe lasers are first combined in a single-mode optical fiber to ensure optimal spatial overlap before being focused through the vapor cell (~50 *μ*m diameter beam waist), after which they are separated by a dichroic beamsplitter. FR fluctuations *θ*_Rb_(*t*) and *θ*_Cs_(*t*) are measured by separate balanced photodiode pairs. The fluctuating output voltages *V_α_*(*t*) (∝ *θ_α_*(*t*), where *α* = Rb, Cs) are continuously digitized and processed in real time. Specifically, we compute the frequency spectrum of the spin noise power density for each species *α*, which is equivalent to the Fourier transform of the spin-spin correlator:
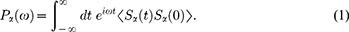


Importantly, we also compute the real part of the cross-correlation spectrum between the Rb and Cs spin fluctuations:
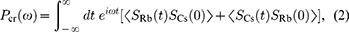
which has not been considered previously for spin noise studies but which, as shown below, contains specific information about inter-species spin coupling and interactions. Note that for clarity, the subscript ‘*z*’ was omitted from all the spin projections *S_z_*(*t*) in Eqs. (1) and (2).

Finally, three orthogonal pairs of large Helmholtz coils are used to cancel out ambient magnetic fields and to apply small (0–5 G) uniform magnetic fields *B_x_* along the transverse 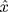 direction. This forces the spin fluctuations *S_z_*(*t*) to precess, thereby shifting the measured spin noise to higher (Larmor) frequencies. The entire setup is constructed of nonmagnetic materials; and magnetic field variations across the 20 mm path length of the probe beams are less than 10^−2^ G.

### Spin noise power spectra

Figure 2(b) shows the power spectra of the detected spin noise from the Rb and Cs spins at *B_x_* = 0 [*P*_Rb_(*ω*) and *P*_Cs_(*ω*); blue and red curves respectively]. The detuning and intensity of the two probe beams were independently adjusted to give approximately equal Rb and Cs spin noise power as measured by the detectors. At *B_x_* = 0 these spin noise peaks are centered at zero frequency and they exhibit approximately Lorentzian lineshapes, indicating exponentially-decaying spin correlations. In principle the linewidth of the spin noise is inversely proportional to the intrinsic relaxation time *τ_s_* of the spin species. However, *τ_s_* is very long in alkali atoms, and so here the observed noise linewidths are additionally broadened in part by the transit-time broadening of the atoms diffusing across the small ~25 *μ*m radius of the focused probe beams (this timescale is of order 10 *μ*s), and also by the fact that comparatively small detunings of the probe lasers were used here in order to obtain large noise signals (*λ*_Rb_ and *λ*_Cs_ were detuned by 28 GHz and 56 GHz from their D1 transitions, respectively). At smaller detunings and/or larger laser intensities, some residual amount of optical pumping will occur which reduces the effective spin lifetimes and broadens the observed spin noise, as was observed and noted in previous spin noise studies of alkali vapors^17^,^30^ and also in solid-state systems^20^.

### Noise cross-correlation spectra

**Figure 3:**
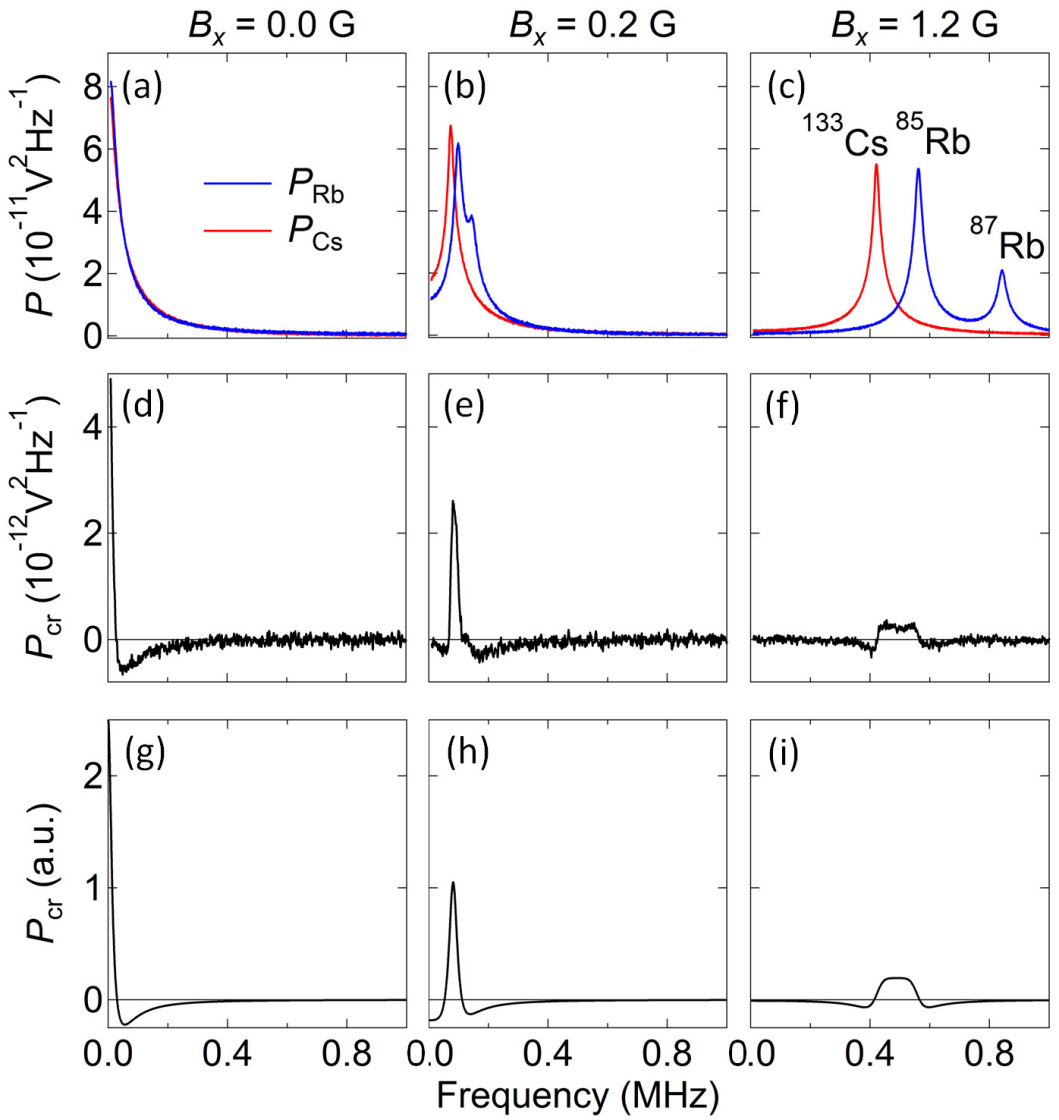
Magnetic field dependence of spin noise power spectra and cross-correlation spectra. (a–c) Measured spin noise power spectra *P*_Rb_(*ω*) and *P*_Cs_(*ω*) at *B_x_* = 0, 0.2, 1.2 G. Here *λ*_Rb_ and *λ*_Cs_ are detuned by 70 GHz and 101 GHz from their respective D1 lines. (d–f) The corresponding cross-correlation spectra *P*_cr_(*ω*). (g–i) Calculation of *P*_cr_(*ω*) [from Eq. (13)], using spin flip rates *γ_A_* = *γ_B_* ≡ *γ*_1_ = 15 kHz, and *γ*_2_ = 60 kHz.

Figure 2(c) shows the corresponding and simultaneously-measured noise cross-correlation spectrum between the two species, *P*_cr_(*ω*). Crucially, *P*_cr_(*ω*) is *not* zero, indicating that *interspecies spin interactions do appear in – and are measurable through – intrinsic spin fluctuations alone*. *P*_cr_(*ω*) exhibits a very narrow peak centered at zero frequency, revealing positive correlations between Rb and Cs spin fluctuations at small frequencies. In addition, *P*_cr_(*ω*) also exhibits a broader negative feature at larger frequencies, revealing *anti*-correlations between Rb and Cs spins at these frequencies. Importantly, *P*_cr_(*ω*) can be fit extremely well by the difference of two Lorentzians with equal area (*i.e.*, *P*_cr_ has zero total integrated area), the origin and significance of which are discussed below. We note that qualitatively similar results are also obtained when using much larger laser detunings, smaller laser intensities, and/or larger beam waists, all of which give narrower noise linewidths [as shown below, *e.g.*, in Fig. 2 (f) and Fig. 3].

To confirm these cross-correlation signals, Figs. 2 (d,e) show similar measurements acquired when one of the probe laser wavelengths is tuned *above* (rather than below) its corresponding D1 transition. While the noise power spectra for the individual vapors are unaffected as expected (because noise power scales as the square of FR; see Eq. (1)), *P*_cr_(*ω*) inverts sign because the FR induced by a polarized ground-state alkali spin is an odd function of wavelength about the D1 transition. That is, a given spin fluctuation will induce a positive- or negative-going FR fluctuation depending on whether the probe laser is red- or blue-detuned from the D1 transition (see inset diagrams). Importantly, it was also verified that cross-correlation signals exist only when the two probe lasers are spatially overlapped in the vapor mixture. Figures 2 (f,g) show that *P*_cr_(*ω*) = 0 when the two probe beams were spatially separated in the vapor, such that they probed Rb and Cs populations that were not directly interacting. Note that in Figs. 2 (f,g), *λ*_Rb_ and *λ*_Cs_ were detuned by 70 GHz and 101 GHz from their D1 transitions, which gives narrower noise linewidths.

### Magnetic field dependence

Figures 3(a–c) show the measured spin noise power spectra from Rb and Cs at different values of *B_x_*. With increasing *B_x_*, the noise peaks shift to higher frequency (due to precession) at different rates in accord with their *g*-factors (1/3 and 1/4, respectively). At 0.2 G the Rb and Cs spin noise peaks are still largely overlapped, while at 1.2 G they are mostly separated. The higher-frequency spin noise peak from the less abundant ^87^Rb isotope (*g* = 1/2) is also visible^12^. Importantly, Figs. 3(d–f) show that the corresponding cross-correlator *P*_cr_(*ω*) also shifts to higher frequencies, diminishes in amplitude, and develops a more complex structure. At larger *B_x_* when the Rb and Cs spin noise peaks no longer overlap at all, *P*_cr_(*ω*) disappears entirely (not shown). Finally, Figs. 3(g–i) show *P*_cr_(*ω*) calculated from the theoretical model that is developed immediately below.

### Sum rule for noise cross-correlators

In order to model and understand these experiments, we develop and apply a theory for interpreting cross-correlations between spin fluctuations in a two-component 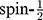 ensemble. We introduce vectors **S***_A_* and **S***_B_* whose components are the total (unnormalized) spin polarization along the *x*, *y*, *z*-axes of type A and B spin in the observation volume. Given numbers *N_Az_*_↑_ and *N_Az_*_↓_ of ↑ and ↓ spins of type A, then *S_Az_* = (*N_Az_*_↑_ − *N_Az_*_↓_)/2. We now formulate a useful sum rule, which is valid irrespective of further model details:

No-Go Theorem: *At thermodynamic equilibrium and in the limit of large spin temperature, the integral of the cross-correlator over frequency is zero*.

Proof: The integral of *P*_cr_(*ω*) over *ω*, defined in (2), gives a delta function in time, which is removed by integration over time to produce a cross-correlator at equal time *t* = 0:
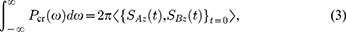
where curly brackets are the anti-commutator. The equilibrium spin density matrix at large temperature is proportional to a unit matrix. The trace of its product with a traceless operator, such as *S_Az_*(*t*)*S_Bz_*(*t*), is zero. Q.E.D.

The no-go theorem shows that, in the limit of large temperatures where *k_B_T* greatly exceeds the energy splittings of available spin states, useful information about interactions between different spin species is contained only in the functional form of *P*_cr_(*ω*). In this limit, it rules out strategies for inferring inter-species spin interactions that are based only on measurements of integrated spin noise power (*e.g.*, Ref. 31). For the case of warm alkali vapors, where *k_B_T* >25 meV but Zeeman and hyperfine energies do not exceed ~10 *μ*eV, the no-go sum rule applies with high accuracy. Hence, it is a valuable tool to test the validity of our theoretical and experimental results.

Finally, we note that the large temperature limit does not mean that the noise power spectrum is trivial. When the temperature scale exceeds dynamic energy scales of the system, all microstates are equally probable. However, this restricts only the distribution of the sizes of spin fluctuations, as it would be revealed by “one-time” measurements of the spin density. In contrast, the two-time spin correlator, as measured with our setup, reveals the *dynamics* of such fluctuations, which is described by the intrinsic spin Hamiltonian. The no-go theorem only introduces a thermodynamic constraint on a single parameter that is the area under the spectral curve. Moreover, this theorem will not hold for ultra-cold atomic gases and many condensed matter systems in which exchange interaction energies exceed the temperature scale. In such situations, the area of *P*_cr_(*ω*) can reveal intrinsic static cross-correlations in the ground state. Another possibility to avoid the restrictions of the no-go sum rule is to consider spin noise in systems that are not in strict thermodynamic equilibrium^32^.

### Theoretical model of spin noise cross-correlators

To model spin interactions and the essential role of spin fluctuations, we first assume that species A and B each have an intrinsic net spin relaxation process with rate *γ_A_* and *γ_B_* per particle (due to, *e.g.*, interactions with cell walls, buffer gas, etc.). To define these rates more quantitatively, we will assume that if species A has polarization *S_Az_* then, on average, this polarization changes by *δS_Az_* = −*γ_A_δtS_Az_* during a small time interval *δt* (and similarly we define a relaxation rate *γ_B_* for species B). In addition, spin-exchange interactions between A and B spins lead to the total-spin-conserving *co-flip processes* with a rate *γ_AB_* per pair of atoms of different kind. On average, each atom of type A can interact with *N_B_*/2 atoms of opposite species, and each atom of type B can exchange spin polarization with *N_A_*/2 of atoms of type *A*, where 1/2 factor follows from the fact that an atom with (say) spin up can exchange spin only with an atom with spin down. With such a definition of kinetic rates, we arrive at the standard evolution equations describing the dynamics of the average spin polarization with spin exchange kinetics:
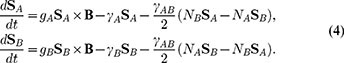


However, in order to study spin fluctuations near thermodynamic equilibrium, Eqs. (4) must be amended to include stochastic fluctuations. It will be convenient to introduce the vector **S** = (**S***_A_*, **S***_B_*)*^T^*, which has components that we will treat as variables having stochastic dynamics. The latter can be written in the form of the following multivariate Langevin equation:
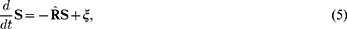
where 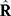 is the *relaxation matrix* with elements 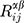, *i*, *j* = *x*, *y*, *z* and *α*, *β* = *A*, *B*, that can be read directly from Eq. (4):
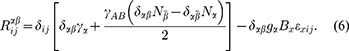
Here we assume that the magnetic field is applied along the *x*-axis and we introduce the bar operator: 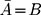 and 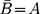. We discuss in the Methods section that, due to the large number of spins in the observation region (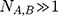) the noise source ***ξ*** can be considered as a white Gaussian noise, *i.e.* its components have correlators
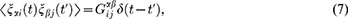
with some coefficients 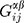 that can be considered as elements of the *correlation matrix* 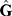. It is the property of the multivariate Langevin equation that in a statistical equilibrium steady state, matrices 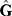 and 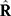 are related by (see, *e.g.* Eq. (4.4.51) in Ref. 33)
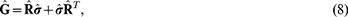
where 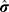 is the *stationary covariance matrix* whose elements are the equal-time correlators of the components of the vector **S**:
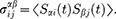
In a general stationary but nonequilibrium stochastic process, Eq. (8) cannot be used to determine 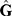 because the matrix 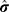, itself, has to be determined by solving Eq. (5). However, at thermodynamic equilibrium the form of the matrix 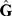 is uniquely determined by the form of the relaxation matrix 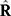 and by the condition that the probabilities of a system's microstates are given by the Boltzmann-Gibbs distribution. This result is generally known as the fluctuation-dissipation theorem.

In our case (the large temperature limit) all spin microstates have equal probabilities. In particular, each spin of each atom can be found with equal probability to have spin projection along the *z*-axis equal to either +1/2 or −1/2, independently of states of other spins at the same moment of time. Since the variance of the sum of independent variables is the sum of individual variances, the covariance matrix elements are
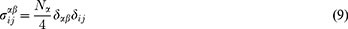
where the Kronecker symbol *δ_αβ_* accounts for the fact that spins of different species can have polarization ±1/2 independently of each other, and *δ_ij_* reflects the fact that the free energy of the system is isotropic. The factor 1/4 in (9) accounts for the fact that the variance of the polarization of a single spin-1/2 is equal to 1/4.

Substituting (6) and (9) into (8) we determine elements of the correlation matrix:
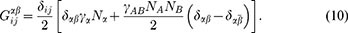


To obtain the cross-correlator, we define 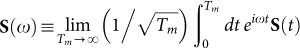, where *T_m_* is the measurement time. By taking the Fourier transform of Eq. (5), and averaging over noise, we obtain spin correlators at the steady state (for a similar approach see also Eq. (4.4.51) in Ref. 33, or Ref. 34):
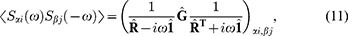
where *i*, *j* = *x*, *y*, *z*; *α*, *β* = *A*, *B*; and 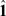 is a unit matrix. The cross-correlator in Eq. (2) is then given by:
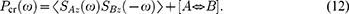
A compact expression can be obtained by assuming identical values for the relaxation rates: *γ_A_* = *γ_B_* ≡ *γ*_1_ (this assumption is not unreasonable for our Rb/Cs mixture).
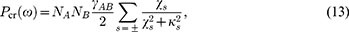
where *χ*_±_ = 2[*γ*_1_*γ*_2_ − (*ω* ± Ω*_A_*)(*ω* ± Ω*_B_*)], *κ*_±_ = 2(*ω* ± Ω*_A_*)Γ*_B_* + 2(*ω* ± Ω*_B_*)Γ*_A_*; Ω*_A_*_,*B*_ ≡ *g_A_*_,*B*_*B_x_* are the Larmor frequencies of the spin species and where
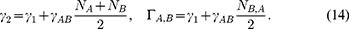
Figures 3(g–i) show *P*_cr_(*ω*) calculated according to Eq. (13). Although the model does not include the less-abundant ^87^Rb isotope, it shows rather excellent agreement with experimental data in Figs. 3(d–f). The calculations used *N_A_* = *N_B_*, which is a reasonable approximation here because *λ*_Rb_ and *λ*_Cs_ were adjusted in the experiment to give equal spin noise power.

## Discussion

We now discuss three different limits of Eq. (13).

**Figure 4:**
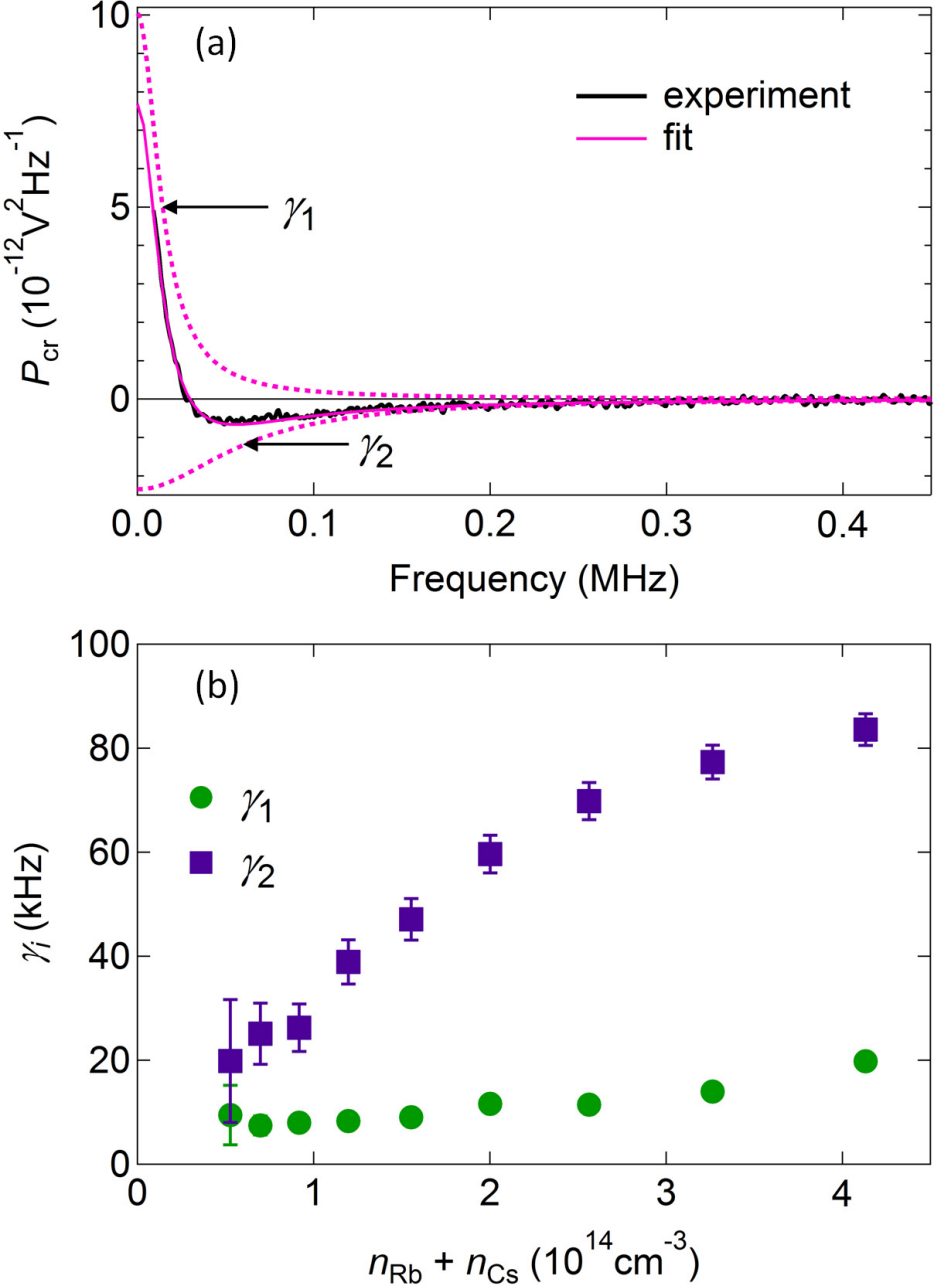
Sum rule for cross-correlators and rates of spin exchange and total spin relaxation. (a) *P*_cr_(*ω*) measured at *B_x_* = 0, fit with two Lorentzians of equal and opposite area (dashed lines), in agreement with the “no-go” theorem. (b) Relaxation rates *γ*_1,2_ extracted from the fit by Eq. (15) versus the total vapor density *n*_Rb_ + *n*_Cs_. Approximately linear dependence is in agreement with the assumption of pairwise spin interactions. The error bars represent *χ*^2^ uncertainty when fitting *P*_cr_(*ω*) to Eq. (15).

- First, at zero applied magnetic field, we find
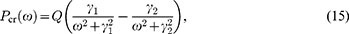
which is simply the difference of two equal-area Lorentzians with widths *γ*_1_ and *γ*_2_. Here *Q* = *N_A_N_B_*/(*N_A_* + *N_B_*).In Fig. 4(a) we show the experimentally measured *P*_cr_(*ω*) at *B_x_* = 0. In good agreement with Eq. (15) and the “no-go” theorem, *P*_cr_(*ω*) has zero total area, being well fit by the difference of two equal-area Lorentzians (dashed lines). Figure 4(b) shows the extracted *γ*_1,2_ as a function of the total vapor density *n*_Rb_ + *n*_Cs_, which is tuned with the cell temperature. Here we recall that *γ*_1_ describes the relaxation of the total spin *S_Az_* + *S_Bz_*. The spin exchange rate is therefore characterized by the difference *γ*_2_–*γ*_1_. As is typical for alkali vapors, we find that the relaxation rate of the total spin is much smaller than the exchange rate, since the latter conserves the total spin. Now we can interpret the negative part of *P*_cr_(*ω*) (15) as emerging from the expected anti-correlations induced by fast spin co-flips between Rb and Cs atoms. On the other hand, the positive-valued peak in (15) and in the data is due to the fact that, at fast co-flip rate, the total spin polarization is equally observed by both beams at longer time scales (*i.e.*, the total spin relaxation is ‘shared’ between the interacting Rb and Cs atoms), which corresponds to positive cross-correlations.
- Next, we consider the limit 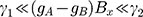, which is close to the case measured in Figs. 3(b,e). Here, one can disregard effects of *γ*_1_ and obtain
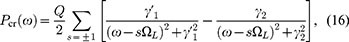
where
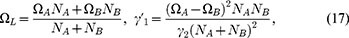
which indicates that the spectrum shifts to an effective (weighted average) Larmor frequency Ω*_L_*, while the positive-valued peak is broadened by the magnetic field (an effective total spin relaxation rate), in agreement with Figs. 3(e,h).
- In the limit of a large magnetic field, Eq. (13) predicts that the cross-correlator *P*_cr_(*ω*) vanishes, also in agreement with experimental observation.

Thus, we see that the theoretical model is confirmed by the experimental data and therefore captures the essential physics of spin fluctuations and their correlations. We note, however, that a more rigorous and quantitative description of the observed noise power and cross-correlations should include not only the presence of all different isotopes but also the coupled dynamics of their nuclear and electronic spins – *i.e.*, the fact that alkali atoms actually have a nontrivial magnetic ground state due to hyperfine splitting – which would lead to multiple correlated resonances even within the same atomic species. On the other hand, our experimental results using widely-detuned lasers apparently validate the simple two-component approximation. This can be explained by the presence of fast intra-species spin-exchange interactions that smear the physics related to presence of multiple resonances from the same atomic species. We also note that, in order to achieve a better theoretical precision, it is straightforward to amend our approach to include multiple intra-species resonances by extending the set of equations (5).

In summary we have shown, both experimentally and theoretically, that cross-correlations between the stochastic spin fluctuations of different species do reveal specific information about spin interactions. Crucially, these interactions can be detected using unperturbed spin ensembles under conditions of strict thermal equilibrium. Such non-invasive characterization techniques may find future applications in metrology, *e.g.* to reveal the physics that limits the efficiency of various magnetometers^18^,^24^. We also envision applications of this technique to mixtures of ultra-cold atomic gases and condensates^35^,^36^, which are sensitive to the probe interference^37^. Studies of cross-correlations of spin noise in solid state physics, *e.g.* in multiple Bloch bands and in new layered materials as well as in artificial semiconductor nanostructures, represent additional and as-yet-unexplored avenues for applications of cross-correlation studies and two-color spin noise spectroscopies.

## Methods

### Microscopic derivation of the correlation matrix

Consider a time interval *δt* much smaller than the ensemble average spin relaxation time but sufficiently large for many random spin flips to happen. The existence of such a time scale is guaranteed by the presence of the large number of atoms in the observation region (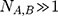). In order to include stochastic fluctuations, we will interpret the kinetic rate, *e.g.* *γ_A_*, in terms of the probability, *p_A_*, for an arbitrary spin of type A to flip per unit of time. The definition of this probability should be consistent with our definition of *γ_A_* in terms of the average relaxation rate. Suppose at time *t* there are *N_A_*_↑_(*t*) atoms with spin +1/2 and *N_A_*_↓_(*t*) with spin −1/2. Since each atom can change its spin by the amount *dS_Az_* = ±1, after a time *δt* the change of the total polarization, on average, will be
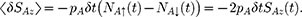
Comparing this with the definition of the relaxation rate, *i.e.* with equation 〈*δS_Az_*(*t*)〉/*δt* = −*γ_A_S_Az_*(*t*), we find that *p_A_* = *γ_A_*/2.

Consider now the variance of the total polarization change due to this process: var(*δS_Az_*) ≡ 〈(*δS_Az_*(*t*))^2^〉 − 〈*δS_Az_*(*t*)〉^2^. If we had only one atom, the variance of spin polarization change would be just *p_A_*(*dS_Az_*)^2^ = *γ_A_δt*/2. The variance of the total change of polarization of *N_A_* atoms is the sum of variances of all independent processes that contribute to it, *i.e.*
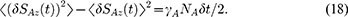
Since the interval *δt* is much smaller than the spin relaxation time, the number of atoms that experience a spin flip during *δt* is much smaller than the total number of atoms. Hence, it is unlikely for any given spin to produce more than one flip in two consecutive time intervals of size *δt*. In turn, this means that spin fluctuations in nearby time intervals are produced essentially by different atoms and can be considered statistically independent. Hence, on a much larger time scale of the spin relaxation, one can assume that spin fluctuations are produced by a white noise, *i.e.* *dS_Az_*/*dt* = −*γ_A_S_Az_* + *η_Az_*(*t*), where 〈*η_Az_*(*t*)〉 = 0, 〈*η_Az_*(*t*)*η_Az_*(*t*′)〉 = *rδ*(*t* − *t*′), and where the coefficient *r* can be obtained by comparing the correlator of the variable 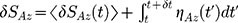 with Eq. (18). This leads us to 〈*η_Az_*(*t*)*η_Az_*(*t*′)〉 = *γ_A_N_A_δ*(*t* − *t*′)/2. Finally, to include fluctuations along different axes, we note that because of the spherical symmetry the equal-time correlator is invariant of coordinate rotation, and such fluctuations must be considered uncorrelated. To describe this situation, we can introduce a vector spin fluctuation source ***η****_A_* such that the total spin polarization vector **S***_A_* changes as *d***S***_A_*/*dt* ~ ***η****_A_*(*t*), where 〈*η_Ai_*(*t*)*η_Aj_*(*t*′)〉 = *δ_ij_γ_A_N_A_δ*(*t* − *t*′)/2; *i*, *j* = *x*, *y*, *z*. Stochastic spin flips of atoms of type *B* are treated similarly, leading to a noise source ***η****_B_* with 〈*η_Bi_*(*t*)*η_Bj_*(*t*′)〉 = *δ_ij_γ_B_N_B_δ*(*t* − *t*′)/2. Obviously, also 〈***η****_A_*(*t*)***η****_B_*(*t*′)〉 = 0.

Next, consider spin-exchange between A and B spins. During time interval *δt*, each atom of type *A* can experience co-flip with one of approximately *N_B_*/2 atoms of type *B*, where the factor 1/2 appears because, on average, only half of the opposite species atoms can participate in the exchange interaction with a given atom.

Each of the *N_A_N_B_*/2 allowed elementary co-flip processes can be considered independent and having a small probability, *p_AB_*, to happen per unit of time. We defined the rate *γ_AB_* so that the average change in *S_Az_*(*t*) during short time interval *δt* due to co-flip processes is then given, up to the linear order in spin polarization, by 〈*δS_Az_*(*t*)〉 = −*γ_AB_δt*[*S_Az_*(*t*)*N_B_* − *S_Bz_*(*t*)*N_A_*]/2. To make the definition of *p_AB_* consistent with this assumption, we should assume that *p_AB_* = *γ_AB_*/2.

If we had only one such a possible process, *i.e.* between only one atom A and one atom B, then a spin of atom A would have a chance to change by ±1 and simultaneously spin of atom B would change by 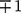. The variance of such a process would correspond to 〈(*dS_Az_*)^2^〉 = 〈(*dS_Bz_*)^2^〉 = (±1)^2^*p_AB_δt* = *p_AB_δt*, and 〈(*dS_Az_*)(*dS_Bz_*)〉 = −*p_AB_δt*. Summing over all independent processes, we find that the variance of total spin polarization fluctuation is given by
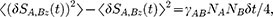

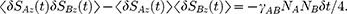
One can check that such correlations can be obtained by introducing a new white noise source *η_ABz_*(*t*) such that 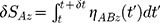 and simultaneously 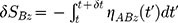, where 〈*η_ABz_*(*t*)*η_ABz_*(*t*′)〉 = *δ*(*t* − *t*′)*γ_AB_N_A_N_B_*/4.

Combining the intrinsic spin dynamics with random inter-species co-flip processes, and introducing external magnetic fields, we find:
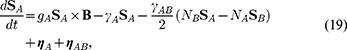

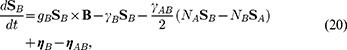
where the noise sources are correlated as
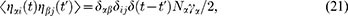

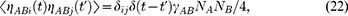
where *i*, *j* = *x*, *y*, *z* and *α*, *β* = *A*, *B*. Eqs. (21)–(22) reproduce the elements of the correlation matrix (10) with
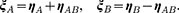
Here we emphasize that the same noise source ***η****_AB_* appears in both equations (19) and (20) with opposite signs. This guarantees the conservation of the total spin at co-flip events. We note that a related model was explored in Ref. 31 in a context different from two-color spin noise spectroscopy. However, instead of a single exchange noise *η_AB_*, two independent noise sources were introduced in Eqs. (19)–(20) in their model. The corresponding system of stochastic equations leads to essentially different predictions and results inconsistent with the no-go theorem, and hence cannot be applied to our case.

## Figures and Tables

**Figure 1:**
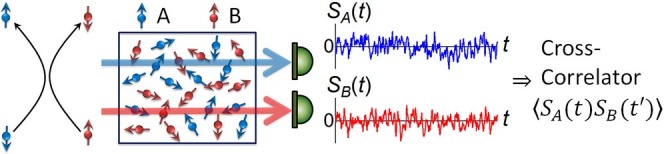
A conceptual experiment wherein spin interactions in a multi-component spin system are revealed via their intrinsic spin fluctuations while in thermal equilibrium. Different probes detect spin fluctuations in the different spin species, A and B. Interactions are revealed via cross-correlations of the two spin noise signals.
